# Supplementary material for: HIVprotI: an integrated web based platform for prediction and design of HIV proteins inhibitors
Source: J Cheminform. 2018 Mar 9;10:12. doi: 10.1186/s13321-018-0266-y (PMC5845081; doi:10.1186/s13321-018-0266-y)
Supplement: Supplementary file 2 — Additional file 2. Source code of HIVProtI web server. [file 13321_2018_266_MOESM2_ESM.zip › HIVprotI_Source-code/hivprotI/weka-3-6-6/doc/weka/experiment/package-frame.html]

weka.experiment


weka.experiment

Interfaces 
  
*Compute*
  
*RemoteExperimentListener*
  
*ResultListener*
  
*ResultProducer*
  
*SplitEvaluator*
  
*Task*
  
*Tester*

Classes 
  
AveragingResultProducer
  
ClassifierSplitEvaluator
  
CostSensitiveClassifierSplitEvaluator
  
CrossValidationResultProducer
  
CSVResultListener
  
DatabaseResultListener
  
DatabaseResultProducer
  
DatabaseUtils
  
DensityBasedClustererSplitEvaluator
  
Experiment
  
InstanceQuery
  
InstancesResultListener
  
LearningRateResultProducer
  
OutputZipper
  
PairedCorrectedTTester
  
PairedStats
  
PairedStatsCorrected
  
PairedTTester
  
PropertyNode
  
RandomSplitResultProducer
  
RegressionSplitEvaluator
  
RemoteEngine
  
RemoteExperiment
  
RemoteExperimentEvent
  
RemoteExperimentSubTask
  
ResultMatrix
  
ResultMatrixCSV
  
ResultMatrixGnuPlot
  
ResultMatrixHTML
  
ResultMatrixLatex
  
ResultMatrixPlainText
  
ResultMatrixSignificance
  
Stats
  
TaskStatusInfo
